# Publisher Correction: TCF1 and LEF1 promote B-1a cell homeostasis and regulatory function

**DOI:** 10.1038/s41586-025-09609-4

**Published:** 2025-09-16

**Authors:** Qian Shen, Hao Wang, Jonathan A. Roco, Xiangpeng Meng, Marita Bosticardo, Marie Hodges, Michael Battaglia, Zhi-Ping Feng, Benjamin James Talks, Jason Powell, Vijaya Baskar Mahalingam Shanmugiah, Julia Chu, Najib M. Rahman, Alguili Elsheikh, Probir Chakravarty, Amalie Grenov, Max Emmerich, Ottavia M. Delmonte, Alexandra F. Freeman, Michael D. Keller, Brahim Belaid, Ilenia Papa, James C. Lee, Pablo F. Cañete, Paula Gonzalez-Figueroa, Yaoyuan Zhang, Hai-Hui Xue, Samra Turajlic, Luigi D. Notarangelo, Muzlifah Haniffa, Lee Ann Garrett-Sinha, Helen M. Parry, Nikolaos I. Kanellakis, Carola G. Vinuesa

**Affiliations:** 1 Francis Crick Institute, London, UK; 2 Division of Immunology and Infectious Disease, John Curtin School of Medical Research, Australian National University, Canberra, Australian Capital Territory Australia; 3 Laboratory of Clinical Immunology and Microbiology, National Institute of Allergy and Infectious Disease, National Institutes of Health, Bethesda, MD USA; 4 School of Infection, Inflammation and Immunology, University of Birmingham, Birmingham, UK; 5 Department of Biochemistry, State University of New York at Buffalo, Buffalo, NY USA; 6 ANU Bioinformatics Consultancy, John Curtin School of Medical Research, Australian National University, Canberra, Australian Capital Territory Australia; 7 Biosciences Institute, William Leech Building, Newcastle University, Newcastle Upon Tyne, UK; 8 Translational and Clinical Research Institute, Newcastle University, Newcastle upon Tyne Hospitals NHS Foundation Trust, Newcastle Upon Tyne, UK; 9 Wellcome Sanger Institute, Wellcome Genome Campus, Cambridge, UK; 10 CAMS Oxford Institute, Nuffield Department of Medicine, University of Oxford, Oxford, UK; 11 Oxford Centre for Respiratory Medicine, Churchill Hospital, Oxford University Hospitals NHS Foundation Trust, Oxford, UK; 12 Renal and Skin Unit, The Royal Marsden Hospital, London, UK; 13 Division of Allergy and Immunology, Children’s National Hospital, Washington, DC USA; 14 Department of Medical Immunology, Beni Messous University Hospital Center, Faculty of Pharmacy, University of Algiers, Algiers, Algeria; 15 Frazer Institute, Faculty of Medicine, The University of Queensland, Brisbane, Queensland Australia; 16 Ian Frazer Centre for Children’s Immunotherapy Research, Child Health Research Centre, Faculty of Medicine, The University of Queensland, Brisbane, Queensland Australia; 17 Centre for Discovery and Innovation, Hackensack University Medical Center, Nutley, NJ USA; 18 CAPTURE Consortium, https://www.royalmarsden.nhs.uk/capture

**Keywords:** Inflammation, Cell biology

Correction to: *Nature* 10.1038/s41586-025-09421-0 Published online 20 August 2025

In the version of this article initially published, plot headings were omitted in Fig. 3a,g, and in the upper and lower plots in Fig. 3g, the current “IL-2–STAT5 signalling” labels were originally written as “IL-5–STAT5 signalling.” The figures have been amended in the HTML and PDF versions of the article.

