# Supplementary material for: TCF1 and LEF1 promote B-1a cell homeostasis and regulatory function
Source: Nature. 2025 Aug 20;646(8084):442–51. doi: 10.1038/s41586-025-09421-0 (PMC12507693; doi:10.1038/s41586-025-09421-0)
Supplement: Supplementary file 1 — Reporting Summary [file 41586_2025_9421_MOESM1_ESM.pdf]

nature portfolio

Corresponding author(s): Carola Vinuesa, Qian Shen

Last updated by author(s): Jun 9, 2025

Reporting Summary

Nature Portfolio wishes to improve the reproducibility of the work that we publish. This form provides structure for consistency and transparency in reporting. For further information on Nature Portfolio policies, see our Editorial Policies and the Editorial Policy Checklist.

Statistics

For all statistical analyses, confirm that the following items are present in the figure legend, table legend, main text, or Methods section.

n/a	Confirmed	
☐	☒	The exact sample size (*n*) for each experimental group/condition, given as a discrete number and unit of measurement
☐	☒	A statement on whether measurements were taken from distinct samples or whether the same sample was measured repeatedly
☐	☒	The statistical test(s) used AND whether they are one- or two-sided<br>*Only common tests should be described solely by name; describe more complex techniques in the Methods section.*
☐	☒	A description of all covariates tested
☐	☒	A description of any assumptions or corrections, such as tests of normality and adjustment for multiple comparisons
☐	☒	A full description of the statistical parameters including central tendency (e.g. means) or other basic estimates (e.g. regression coefficient) AND variation (e.g. standard deviation) or associated estimates of uncertainty (e.g. confidence intervals)
☐	☒	For null hypothesis testing, the test statistic (e.g. *F*, *t*, *r*) with confidence intervals, effect sizes, degrees of freedom and *P* value noted<br>*Give P values as exact values whenever suitable.*
☒	☐	For Bayesian analysis, information on the choice of priors and Markov chain Monte Carlo settings
☒	☐	For hierarchical and complex designs, identification of the appropriate level for tests and full reporting of outcomes
☐	☒	Estimates of effect sizes (e.g. Cohen's *d*, Pearson's *r*), indicating how they were calculated

Our web collection on statistics for biologists contains articles on many of the points above.

Software and code

Policy information about availability of computer code

Data collection	TCF1 and LEF1 RNA expression in mouse B cell subsets were collected from ImmGen. Cellular phenotype data was collected on a Fortessa, Fortessa X-20, LSR II or FACSAria II and software (CellQuest and FACSDiva version 8.0 and 9.2; BD). ELISA data was collected by using an Infinite® 200 PRO plate reader (Tecan) equipped with i-control™ version 1.9 software. RNA-seq data was collected on a HiSeq2000 machine with a depth of more than 30 million reads per samples. scRNA-seq data was collected using the NovaSeq 6000 (Illumina) system or NovaSeq S2. scRNA-seq on human prenatal B-1 were downloaded from the developmental cell atlas portal (https://developmentcellatlas.cellgeni.sanger.ac.uk/fetal-immune/lymphoid/). ChIP-seq data were downloaded from NCBI's GEO database for TCF1 and LEF1 CHIP-seq data (SRP142342).ATAC-seq data were downloaded from NCBI's GEO database for ATAC-seq samples in B-1a cells (GSM2461745). Immunohistochemistry images were collected on the PhenoImager HT (Akoya Biosciences).
Data analysis	Comparison between groups was performed using parametric t test, two-tailed Welch's t test or Mann-Whitney t test, one way ANOVA with Tukey multiple-comparison test, two-way ANOVA, two-tailed Pearson correlation analysis from GraphPad Prism10 (GraphPad Software, USA). Sample sizes are provided in the figures and statistically significant differences are indicated as exact P value. Flow cytometry data was analysed using the Flowio software v10 (Flowio LLC). RNA-seq analysis were aligned to the mm10 (GRC38) genome assembly using hisat2 and the mapped reads were assigned with FeatureCounts [v2.4]. Differential expression analyses were performed with voom-limma, after removal of lowly expressed genes and normalized using the trimmed mean of M-values method. Significantly differentially expressed genes were identified by applying a Benjamini–Hochberg adjusted P value threshold of 0.05. Gene set enrichment or pathway analysis were performed using clusterProfiler and Camera against the gene ontology (GO) database, KEGG database and HALLMARK, C2 and C7 gene sets in the MSigDB (v7.5). scRNA-seq data was analysed using Cell Ranger pipeline v.6.0.1 or v7.0.1. Data were processed and analysed using the Seurat package (v5.0.3) in R (v4.4.1). Raw gene expression matrices were filtered to exclude low-quality cells based on thresholds for mitochondrial gene content (set at 5%) and the number of detected genes per cell, using the PercentageFeatureSet() and subset() functions. For multiplexed samples labelled with cell hashing antibodies, demultiplexing was performed using the MULTIseqDemux() function in Seurat.

Hashtag oligonucleotide (HTO) counts were normalised using centred log-ratio (CLR) transformation via the NormalizeData() function with normalisation.method = "CLR", and cells were classified as singlets, doublets, or negatives based on HTO signal. Only singlets were retained for downstream analysis. Normalisation and integration were performed using the scTransform() workflow, followed by the identification of highly variable features. Dimensionality reduction was performed using principal component analysis (PCA) via the RunPCA() function, and the top 20–30 principal components were selected to construct a shared nearest neighbour (SNN) graph using FindNeighbours(). Clustering was conducted using the FindClusters() function. Clusters were visualised using Uniform Manifold Approximation and Projection (UMAP) via the RunUMAP() function. Differential expressed genes for each cluster were identified using the FindAllMarkers() function with test.use set as either "wilcox" or "MAST". Where applicable, cell types were annotated based on canonical marker expression or informed by publicly available datasets. Cell trajectory analysis was performed using Monocle2 v2.32.0 in R v4.4.1. The annotated Seurat object was first transformed into a CellDataSet object using the as.CellDataSet() function from the Seurat package. To filter relevant genes, those expressed in at least 10 cells and with a mean expression value of 0.1 or greater were selected. Next, highly variable genes across cell subsets were identified using the differentialGeneTest() function with the formula fullModelFormulaStr = '~cell_subset'. Based on their q-value, the top 2000 genes were selected, and setOrderingFilter() was applied to prioritize them for ordering in pseudo time analysis. Dimensionality reduction was performed using reduceDimension() with the parameter reduction_method = 'DDRTree'. Cells were subsequently ordered along the trajectory using the orderCells() function and visualized with plot_cell_trajectory().B-cell receptor (BCR) repertoire analysis was conducted with the Immcantation framework, following the guidelines provided by its developers (http://immcantation.org/). This was implemented in a Python environment (v.3.11.10). We began with 10x Genomics BCR sequencing data processed through the CellRanger pipeline (v7.0.1) to obtain annotated clonotype and contig sequences. For V(D)J gene assignment, the AssignGenes.py command from the Change-O toolkit was employed, referencing the mouse IMGT and IgBLAST (v1.22.0) databases. MakeDb.py was used to standardize the data to AIRR format. Clonotypes were assigned using the DefineClones.py tool based on heavy chain sequences, employing the default Hamming distance substitution model. Subsequently, light_cluster.py was applied to refine clonotype grouping by incorporating light chain information. Finally, the resulting datasets were merged with the annotated Seurat file and key metrics, including V(D)J gene usage, CDR and FWR sequences, lengths, and mutational content were extracted for downstream analysis and visualization.Cell trajectory analysis was performed using Monocle2 v2.32.0 in R v4.4.1. The annotated Seurat object was first transformed into a CellDataSet object using the as.CellDataSet() function from the Seurat package. To filter relevant genes, those expressed in at least 10 cells and with a mean expression value of 0.1 or greater were selected. Next, highly variable genes across cell subsets were identified using the differentialGeneTest() function with the formula fullModelFormulaStr = '~cell_subset'. Based on their q-value, the top 2000 genes were selected, and setOrderingFilter() was applied to prioritize them for ordering in pseudo time analysis. Dimensionality reduction was performed using reduceDimension() with the parameter reduction_method = 'DDRTree'. Cells were subsequently ordered along the trajectory using the orderCells() function and visualized with plot_cell_trajectory(). B-cell receptor (BCR) repertoire analysis was conducted with the Immcantation framework, following the guidelines provided by its developers (http://immcantation.org/). This was implemented in a Python environment (v.3.11.10). We began with 10x Genomics BCR sequencing data processed through the CellRanger pipeline (v7.0.1) to obtain annotated clonotype and contig sequences. For V(D)J gene assignment, the AssignGenes.py command from the Change-O toolkit was employed, referencing the mouse IMGT and IgBLAST (v1.22.0) databases. MakeDb.py was used to standardize the data to AIRR format. Cells expressing more than one heavy chain or only light chains were removed. Clonotypes were assigned using the DefineClones.py tool based on heavy chain sequences, employing the default Hamming distance substitution model. Subsequently, light_cluster.py was applied to refine clonotype grouping by incorporating light chain information. Finally, the resulting datasets were merged with the annotated Seurat file and key metrics, including V(D)J gene usage, CDR and FWR sequences, lengths, and mutational content were extracted for downstream analysis and visualization.For diversity analysis, we used the iNEXT package (v3.0.1) to perform coverage-based rarefaction and extrapolation analyses. We computed the three most common Hill numbers: species richness (q = 0), Shannon diversity (q = 1) and Simpson diversity (q = 2). The resulting sampling curves, which illustrate diversity estimates with respect to sample coverage, were visualized using ggiNEXT() function. Additionally, we constructed circle packing plots using the packcircles package (v0.3.6) to visualize the immune repertoire composition of individual samples. These analyses were conducted in R v4.4.1.FASTQ files were aligned to Enembl's mouse GRCm38 genome using BWA version 0.7.15. The resulting BAM files were sorted, duplicates marked and indexed using Picard version 2.1.1. Peaks were called using MAC2 version 2.1.1 that were enriched in TCF1 or LEF1 relative to input using default parameters. Peaks were annotated using homer version 4.8. BAM files were normalised to 10 million reads and IGVTools version 2.3.75 was used to generate coverage files. Slides were counterstained with DAPI and imaged on the PhenoImager HT (Akoya Biosciences).

For manuscripts utilizing custom algorithms or software that are central to the research but not yet described in published literature, software must be made available to editors and reviewers. We strongly encourage code deposition in a community repository (e.g. GitHub). See the Nature Portfolio guidelines for submitting code & software for further information.

Data

Policy information about availability of data

All manuscripts must include a data availability statement. This statement should provide the following information, where applicable:

- Accession codes, unique identifiers, or web links for publicly available datasets
- A description of any restrictions on data availability
- For clinical datasets or third party data, please ensure that the statement adheres to our policy

The authors declare that data supporting the findings of this study are available within the paper or its supplementary information. Datasets that support the findings of this study are accessible at the following repositories: RNA-seq data (GSE290505), scRNA-seq and scBCR-seq data (GSE294717 and GSE298030). ChIP-seq and ATAC-seq data are obtained from publicly available dataset (SRP142342 and GSM2461745). Processed and annotated single cell RNA sequencing data of foetal immune cells, specifically the HSPCs and the B cell lineages, in Suo et al, Science 2022 23 were downloaded from the developmental cell atlas portal (https://developmentcellatlas.cellgeni.sanger.ac.uk/fetal-immune/lymphoid/).

Research involving human participants, their data, or biological material

Policy information about studies with human participants or human data. See also policy information about sex, gender (identity/presentation), and sexual orientation and race, ethnicity and racism.

Reporting on sex and gender	The study involves both male and female participants.

Reporting on race, ethnicity, or other socially relevant groupings	This study does not include any variables on race, ethnicity, or other socially relevant groups.
Population characteristics	Individuals were either healthy controls, or patients who were diagnosed with bacterial pleural infection, chronic lymphocytic leukaemia (CLL), severe combined immunodeficiency (SCID), Otitis media with effusion (OME) and sleep disordered breathing (SDB) by treating physicians Individuals known medical treatments and clinical diagnosis are provided in Extended Table 1-3
Recruitment	Participants were recruited by their referring medical practitioners:<br>1.Pleural infection specimens: Patients with confirmed bacterial pleural infection were invited to donate samples (Extended Table 1); all patients had pleural neutrophil counts > 10 million/L and neutrophil/lymphocyte ratio (NLR) >4 and were on antibiotic treatment. All clinical specimens (pleural fluid and blood) were collected for Oxford Radcliffe Pleural Biobank .<br>2. CLL patients and healthy donors were also recruited following favourable study approval on 04Mar2024 by the London-Brent Research Ethics Committee and as part of the CAPTURE (NCT03226886) study, a prospective longitudinal cohort study investigating Covid vaccine responses in cancer patients (Extended Table 2).<br>3.SCID patients and healthy donors were obtained by by the Institutional Review Boards of Children's National Hospital (Washington DC) and of the National Institute of Health (Bethesda, MD) .<br>4.Adenoid tissue samples were collected from children (aged 2 — 6 years, 2 females and 4 males) undergoing elective adenoidectomy surgery for either otitis media with effusion (OME) or sleep disordered breathing (SDB) at the Great North Children's Hospital, Newcastle Upon Tyne, UK.
Ethics oversight	The study was approved by and complies with all relevant ethical regulations of:<br>1. All clinical specimens (pleural fluid and blood) were collected for Oxford Radcliffe Pleural Biobank (Ethical approval reference: 19/SC/0173, South Central - Oxford C Research Ethics Committee) and the study was approved by the Oxford Radcliffe Biobank Tissue Access Committee (reference number: 22/A093).<br>2. All CLL patients were approved by favourable study approval on 04Mar2024 by the London- Brent Research Ethics Committee (REC Reference 24/PR/0218, IRAS 330505) and as part of the CAPTURE (NCT03226886) study, a prospective longitudinal cohort study investigating Covid vaccine responses in cancer patients. CAPTURE is sponsored by The Royal Marsden Hospital and was approved as a substudy of TRACERx Renal (NCT03226886) by the National Research Ethics Service Committee London, Fulham on 01/05/2020 (REC 11/LO/1996).EXACT is sponsored by the Royal Marsden Hospital and was approved by the National Research Ethics Service Committee, West Midlands - Black Country on 22/11/2021 (REC/WM/0251).<br>3. Blood fro SCID patients is approved by the Institutional Review Boards of Children's National Hospital (Washington DC) and of the National Institute of Health (Bethesda, MD) and 93-1-0119, approved by the Institutional Review Board of the National Institutes of Health (Bethesda, MD).<br>4. Adenoid tissue samples were approved by Newcastle University biobank (project code: NRTB-1).<br>5. Ethical approval to obtain blood from healthy individuals was provided by the London-Brent Regional Ethics Committee (REC:21/LO/0682).

Note that full information on the approval of the study protocol must also be provided in the manuscript.

Field-specific reporting

Please select the one below that is the best fit for your research. If you are not sure, read the appropriate sections before making your selection.

☒ Life sciences ☐ Behavioural & social sciences ☐ Ecological, evolutionary & environmental sciences

For a reference copy of the document with all sections, see nature.com/documents/nr-reporting-summary-flat.pdf

Life sciences study design

All studies must disclose on these points even when the disclosure is negative.

Sample size	For each experiment we estimated the expected change between experimental and control groups (e.g. at least a 20% change and SD at most half the magnitude of the minimum effect size we were interested in). With those assumptions we used power analysis to estimate the group size that would provide at least 80% power to detect statistically-significant difference (with p < 0.05 considered significant).
Data exclusions	No data was excluded from the analysis
Replication	Fig 1a No experimental replication was feasible<br>Fig 1b-d are representative of 2 experiments<br>Fig 1e-f are representative of 5 experiments<br>Fig 1g are 3 patients (pleural infection), 11 patients (CLL patients), 7 healthy donors, 6 patients (SDB/OME).<br>Fig 1h is representative of 3 healthy donors<br>Fig.1i is representative for 11 patients<br>Fig.1j-m are representative for 3 experiments<br>Fig.1n is representative for 4 experiments<br>Fig 2a-d are representative of 2 experiments<br>Fig 2e-f No experimental replication were feasible<br>Fig 2g-h are representative of 2 experiments<br>Fig 3a No experimental replication is feasible

Fig 3b is representative of 2 experiments
Fig 3c-h No experimental replication are feasible .
Fig 4a-b No experimental replication is feasible.
Fig 4c-h are representative of 2 experiments.
Fig 4i-j are representative of 2 experiments.
Fig 4k is representative of 2 experiments.
Fig 41-n are representative of 2 experiments.
Fig 4o are representative of 3 mice.
Fig 5a-k are representative of 2 experiments.
Fig 5l No experimental replication is feasible
Extended Fig 1a-b No experimental replication was feasible.
Extended Fig 1c-g are representative of 2 experiments
Extended Fig 1h is representative of 3 patients (pleural infection)
Extended Fig 1i is representative of 3 patients (pleural infection) and 6 patients (SDB /OME)
Extended Fig 1j is representative of 6 healthy donors
Extended Fig 1k is representative of 3 healthy donors
Extended Fig 2a-c is representative of 3 experiments
Extended Fig 2d-e is representative of 4 experiments
Extended Fig 3a No experimental replication was feasible.
Extended Fig3b-i are representative of 2 experiments
Extended Fig 4a-c are representative of 2 experiments
Extended Fig 5a-b No experimental replication was feasible
Extended Fig 5c pooled data from 3 experiments
Extended Fig 5d are representative of 3 experiments
Extended Fig 5e is either representative of 3 experiments (top) or pooled data from 3 experiments (bottom)
Extended Fig 5f is representative of 3 experiments
Extended Fig 6a-f No experimental replication was feasible
Extended Fig 6g is representative of 7 donors.
Extended Fig 6h No experimental replication was feasible
Extended Fig 7a is representative of 2 experiments
Extended Fig 7b-c are representative of 2 experiments
Extended Fig 7d-f No experimental replication was feasible
Extended Fig 7g is representative of 2 experiments
Extended Fig 7h is representative of 5 mice.
Extended Fig 7i-k are representative of 2 experiments
Extended Fig 7l is No experimental replication was feasible

Randomization

For in vitro experiments, randomization was not required given there were no relevant covariates (i.e. cells from littermate mice came from the same cage, all wells treated simultaneously using multi-channel pipettes, on the same day, in the same single plate, analysed in the same machine, handled by the same investigator). This was not relevant to the analysis of human PBMC samples, as no experimental interventions were undertaken on these samples.

Blinding

Investigators planned mouse experiments based on genotype and grouping, but during performance of experiments mice were identified only by randomly assigned number with investigators blind to group allocation. This was not relevant to our human study, as group allocation was not based on any interventions, but the clinical phenotype of human sample donors. CLL samples in particular were impossible to blind given their composition of predominantly malignant cells, which are immediately apparent in cytometric analysis.

Reporting for specific materials, systems and methods

We require information from authors about some types of materials, experimental systems and methods used in many studies. Here, indicate whether each material, system or method listed is relevant to your study. If you are not sure if a list item applies to your research, read the appropriate section before selecting a response.

Materials & experimental systems

n/a	Involved in the study	
☐	☒	Antibodies
☒	☐	Eukaryotic cell lines
☒	☐	Palaeontology and archaeology
☐	☒	Animals and other organisms
☒	☐	Clinical data
☒	☐	Dual use research of concern
☒	☐	Plants

Methods

n/a	Involved in the study	
☐	☒	ChIP-seq
☐	☒	Flow cytometry
☒	☐	MRI-based neuroimaging

Antibodies

Antibodies used

All antibodies used are commercially availiable and extensively used. We have listed all antibodies and their clone names in the materials section but given the large number of antibodies used over the breadth of the work we did not note all their lot numbers. B220-BUV737 (RA3-6B2, BD Horizon #612838), CD19-BV605(6D5, Biolegend #115540), CD23-BV421(B3B4, Biolegend #101621), CD5-APC (53-7.3,ebioscience #17-0051-82), IgM-PE-Cy7 (II/41,ebioscience, #25-5790-82), CD19-BUV395(1D3, BD Horizon, #563557), CD3-

	BV650 (17A2, Biolegend, #100229), CD21-BV605(7G6, BD #747763), IgD-PerCP/Cyanine5.5(11-26c.2a, Biolegend, #405710), CD93-BV480(AA4.1, BD, #746239), CD93-PE/Cyanine7(AA4.1, Biolegend, #136506), CD24-Pacific Blue(M1/69, Biolegend #101820), CD43-BV605(S7, BD, #747726), CD43-BV711 (S7, BD, #740668), CD3-APC-Cy7(17A2,Biolegend #100222), CD4-APC-Cy7(GK1.5, Biolegend, #100414), CD11b-APC-Cy7(M1/70, Biolegend,#101226), CD11b-AF700(M1/70,Biolegend,#101222), TER119-APC-Cy7(TER-119, BD, #560509), Gr1-APC-Cy7(RB6-8C5, Biolegend, #108424), Sca-1-BV421(Ly-6A/E,Biolegend, # 108128), c-Kit-APC(2B8, Biolegend, #105812), CD127-PE-Cy7(A7R34, Biolegend, #135014), CD16/32-PerCP/Cyanine5.5(93, Biolegend, #101324), CD135-PE(A2F10, Biolegend, #135306), CD45.1-BV711(A20, Biolegend, #110739), CD45.2-BUV737 (104, BD, #612779), CD274-BV711(B7-H1, Biolegend, # 124319), CD86-PE-Cy7(GL-1, Biolegend, #105014), FCRL5-AF488(biotechne, FAB6757G), BLNK phosphorylated at Tyr84 (J117-1278, BD, #558442), Erk1/2 phosphorylated at Thr202/Tyr204 (20A, BD, #561991), phosphorylated PLCγ2 (K86-1161, BD, #560134), Syk phosphorylated at Tyr525/526 (C87C1, Cell Signaling Technology, #6485S) and Btk phosphorylated at Tyr551 (M4G3LN, eBioscience, #12-9015-42), c-Myc-AF647(Y69, abcam, ab190560), TCF1/TCF7-AF488(C63D9, Cell Signaling Technology, #6444S),LEF1-AF488(C12A5, Cell Signaling Technology, #8490S), DOPC/CHOL/Fluorescein-DHPE (54:45:1); Rhodamine-DH PE Liposomes(DOPC/CHOL/Rhodamine-DHPE(54:45:1) were used (FormumMax, Sunnyvale,CA, F60103F2-R). IL10-PE (JES5-16E3, Biolegend, #505008), IL10-BV421 (JES5-16E3, BD, #563276), CD223(LAG-3)-PE(C9B7W, Biolegend, #125208), CD138-BV605(281-2, Biolegend, #142515),CD138-PE(281-2, Biolegend, #142504), CD43-BV711 (S7, BD, #740668), CD1d-PerCP/Cy5.5 (1B1, Biolegend, #123513), Ki-67-PerCP-eFluorTM710 (SolA15, eBioscience, #46-5698-82), Ig light chain κ-AF700 (RMK-45, Biolegend, #409508),Ig light chain λ-FITC (R26-46, BD,#553434), IgG3-Biotin (RMG3-1, Biolegend, #406803); For human sample staining: TruStain FcX CD16/32/64 antibodies (Biolegend, #422302), CD3-BV785 (HIT3a, BD Bioscience,#740961), CD19-BUV737(SJ25C1, BD Bioscience, #612756), CD19-PerCP/Cyanine5.5 (SJ25C1, Biolegend, #363016), CD27-PECy7(M-T271, BD Bioscience, #560609), CD38-BV605 (HIT2, BD Bioscience, #569699), CD43-BV510(1G10, BD Horizon, #563377), CD43-FITC(1G10, BD, #555475), CD38-BV510 (HIT2, Biolegend, #303540), CD5-APC (UCHT2, Biolegend, #300612), CD5-PE/Cyanine7 (UCHT2, Biolegend, #300622), IgA-PerCP-Vio700 (IS11-8E10, MACS, #130-113-478), CD24-BV711 (ML5, BD, #563401), IgM-BUV395 (G20-127, BD, #563903), TCF1/TCF7(C63D9, Cell Signaling Technology,#2203), LEF1(EPR2029Y, Abcam. #ab137872), Alexa Fluor 488 Donkey anti-rabbit IgG (minimal x-reactivity Poly4064, Biolegend, #406416), Alexa Fluor 647 Donkey anti-rabbit IgG (minimal x-reactivity Poly4064, Biolegend, #406414), Streptavidin-BV605 (Biolegend, #405229).
Validation	All antibodies used were commercial antibodies and had been previously validated by the manufacturing companies. We provide the clones used for each antibody. Antibody titrations and dilutions used in each experiment are only relevant to the specific batch used, which change over time and therefore not useful.

Animals and other research organisms

Policy information about studies involving animals; ARRIVE guidelines recommended for reporting animal research, and Sex and Gender in Research

Laboratory animals	C57BL/6 mice were used in this study. Both male and female mice were used. Mice were used at 8-12 weeks for phenotyping and in vitro experiments, except for aging assessment (4-21 weeks). The animals are held within a Techniplast IVC green line system, the air movement in the cages is regulated by an Techniplast air management unit on negative pressure, 75 ACH/-20%, and all cages are placed on automatic watering, which is chlorinated to 2.45%. The humidity and temperature are regulated at room level, set at code of practice standard levels, 20-24C and 55% +/- 10%. The light cycle is 7am-7pm including dawn and dusk settings of 15 minutes (6:45-7 am and pm). The animals are kept on Datesand Eco Pure Chips sawdust, Bed'r'Nest nesting and smart homes enrichment, with Teklad Global Rodent Diet Sterlised 2018S 18% Protein.
Wild animals	The study did not involve wild animals
Reporting on sex	The study involves both male and female.
Field-collected samples	The study did not involve field animals.
Ethics oversight	Animal experimentation was performed according to the regulations approved by UK Home Office under project license (PP2867252),Australian National University's Animal Experimentation Ethics Committee and University at Buffalo,

Note that full information on the approval of the study protocol must also be provided in the manuscript.

Plants

Seed stocks	The study did not involve plants.
Novel plant genotypes	The study did not involve plants.
Authentication	The study did not involve plants.

ChIP-seq

Data deposition

☒ Confirm that both raw and final processed data have been deposited in a public database such as GEO.

☒ Confirm that you have deposited or provided access to graph files (e.g. BED files) for the called peaks.

Data access links *May remain private before publication.*	FASTQ files were downloaded from NCBI's GEO database for TCF1 CHIP-seq data (SRP142342)
Files in database submission	This study analyzed published ChIP data (SRP142342)
Genome browser session (e.g. UCSC)	This analysis doesn't have Genome browser session.

Methodology

Replicates	2 replicates
Sequencing depth	Information at SRP142342
Antibodies	TCF1
Peak calling parameters	Peaks were called using MAC2 version 2.1.1 that were enriched in TCF1 relative to input using default parameters. Peaks were annotated using homer version 4.8.
Data quality	FASTQ files were aligned to Enembl's mouse GRCm38 genome using BWA version 0.7.15. The resulting BAM files were sorted, duplicates marked and indexed using Picard version 2.1.1. Peaks were called using MAC2 version 2.1.1 that were enriched in TCF1 relative to input using default parameters. Peaks were annotated using homer version 4.8. BAM files were normalised to 10 million reads.
Software	IGVTools version 2.3.75 was used to generate coverage files.

Flow Cytometry

Plots

Confirm that:

☒ The axis labels state the marker and fluorochrome used (e.g. CD4-FITC).

☒ The axis scales are clearly visible. Include numbers along axes only for bottom left plot of group (a 'group' is an analysis of identical markers).

☒ All plots are contour plots with outliers or pseudocolor plots.

☒ A numerical value for number of cells or percentage (with statistics) is provided.

Methodology

Sample preparation	Pleural fluid and blood specimens were transferred and processed in the lab within hours after collection. Pleural fluid specimens were centrifuged at 800g for 10 minutes. The acellular supernatant was removed and stored in -80oC. Then the cellular pellet was resuspended in red blood cell lysis buffer (5-10 ml, J62990.AK Thermoscientific) and incubated for 5 minutes. The sample was centrifuged at 500g for 5 minutes and the supernatant was discarded. If there were red blood cells the process was repeated, otherwise the cells were washed in 5 ml of PBS and centrifuged at 500g for 5 minutes. The supernatant was discarded and the cells were resuspended in 5ml of RPMI enriched with 10% FBS, passed through a 70um filter and counted. Whole blood was collected in EDTA tubes (VWR) and stored at 4°C until processing or processed freshly. All samples were processed within 24 hours. Prior to processing, tubes were brought to room temperature (RT). PBMC and plasma were isolated by density-gradient centrifugation using centrifugation tubes (SepMateTM, STEMCELL) per manufacturer's instructions. PBMCs were either proceed to be stained with antibodies directly or resuspended in Recovery cell culture freezing medium (Fisher Scientific) containing 10% DMSO, placed overnight in CoolCell freezing containers (Corning) at -80°C and then transferred to liquid nitrogen for longtime storage. Adenoid tissue samples were collected in phosphate buffered solution and immediately mechanically dissociated into a single cell suspension using scalpel blades and a 100 µm filter. Cells were then cryopreserved in freezing media (10% DMSO and 90% FBS) in liquid nitrogen prior to flow cytometry experiments.<br>Adenoid tissue samples were collected in phosphate buffered solution and immediately mechanically dissociated into a single cell suspension using scalpel blades and a 100 µm filter. Cells were then cryopreserved in freezing media (10% DMSO and 90% FBS) in liquid nitrogen prior to flow cytometry experiments.<br>Single cell suspensions were prepared from mice peritoneal cavity and spleens and B-1 cells and B-2 cells were magnetically purified using mouse B Cell Isolation Kit (Miltenyi Biotec) with anti-mouse CD45R (B220) antibody (Miltenyi Biotec, 130-110-707), labeled with Cell Trace Violet (CTV, Thermo Fisher) and cultured for<br>72 hours in complete RPMI 1640 media (Sigma-Aldrich) supplemented with 2mM L-Glutamine (GIBCO), 100 U penicillinstreptomycin (GIBCO), 0.1 mM nonessential amino acids (GIBCO), 100 mM HEPES (GIBCO), 55 mM 0-

mercaptoethanol (GIBCO) and 10% FBS (GIBCO) followed by stimulating with or without 5µg ml-1 LPS (O111:B4, Sigma, L4391), 0.4 µg ml-1 IL-10 (Thermo Fisher, 210-10-10UG), 10 µg ml-1 InVivoMAb anti-mouse IL-10R (CD210) (BioXCell, BE0050) and its isotype control, InVivoMAb rat IgG1 isotype control, anti-horseradish peroxidase (BioXCell, BE0088) at 37°C in 5% CO_2. Peritoneal B-1a cells are obtained from mice, the Fc receptors blocked and cells stained and sorted.
For cytokine intraceullular staining, the cells were stimulated with PMA, ionomycin and brefeldin A for the terminal 5h of culture. Cells were harvested and stained for surface markers, including ebioscience Fixable eFluor780 viability dye (Invitrogen) to exclude dead cells before cells were fixed. Intracellular staining was performed with Cytofix/Cytoperm kit (BD) with IL10-PE (JESS-16E3, Biolegend) as recommended. For transcription factors, eBioscience Foxp3/transcription factor staining buffer set (Invitrogen) was used per manufacturer's instructions.

Instrument

Cells were sorted on a FACS Aria II, splenocytes and human PBMC samples were acquired on a Fortessa or Fortessa X-20 cytometer.

Software

FACS data was analyzed using FlowJo software v10.10.0 (FlowJo LLC).

Cell population abundance

Sorted sample purity was based on flow cytometry sorting analysis and stringent gating. Abundance of populations are indicated in the gating figures of the manuscript.

Gating strategy

SC-H/FSC-A (cells were gated along a diagonal gating strategy to eliminate cells with disproportional FSC-H and FSC-A size), SSC-W/SSC-H (cells with large SSC-W from scatter were eliminated), FSC-A/Live dead (cells staining negative for the live dead marker were selected as "live") and FSC-A/SSC-A (Cells were gated as lymphocytes if they had a lower size and granularity relative to other signals detected). Once cells were established as singlets, live and lymphocytes analysis was completed as described in the manuscript, where possible biphasic populations were used to identify positive and negative populations.

☒ Tick this box to confirm that a figure exemplifying the gating strategy is provided in the Supplementary Information.
